# Electrochemical Quantification of H_2_O_2_ Released by Airway Cells Growing in Different Culture Media

**DOI:** 10.3390/mi13101762

**Published:** 2022-10-18

**Authors:** Bernardo Patella, Serena Di Vincenzo, Claudio Zanca, Luciano Bollaci, Maria Ferraro, Maria Rita Giuffrè, Chiara Cipollina, Maria Giuseppina Bruno, Giuseppe Aiello, Michele Russo, Rosalinda Inguanta, Elisabetta Pace

**Affiliations:** 1 Department of Engineering, University of Palermo, 90128 Palermo, Italy; 2 Institute of Traslational Pharmacology (IFT), National Research Council of Italy (CNR), 90146 Palermo, Italy; 3 Ri.MED Foundation, 90146 Palermo, Italy; 4 Dipietro Group, 96010 Melilli, Italy

**Keywords:** H_2_O_2_, electrochemical sensor, cell culture media, graphene oxide, gold, bronchial epithelial cell, lung adenocarcinoma cell, oxidative stress, cigarette smoke extract, resveratrol

## Abstract

Quantification of oxidative stress is a challenging task that can help in monitoring chronic inflammatory respiratory airway diseases. Different studies can be found in the literature regarding the development of electrochemical sensors for H_2_O_2_ in cell culture medium to quantify oxidative stress. However, there are very limited data regarding the impact of the cell culture medium on the electrochemical quantification of H_2_O_2_. In this work, we studied the effect of different media (RPMI, MEM, DMEM, Ham’s F12 and BEGM/DMEM) on the electrochemical quantification of H_2_O_2_. The used electrode is based on reduced graphene oxide (rGO) and gold nanoparticles (AuNPs) and was obtained by co-electrodeposition. To reduce the electrode fouling by the medium, the effect of dilution was investigated using diluted (50% *v*/*v* in PBS) and undiluted media. With the same aim, two electrochemical techniques were employed, chronoamperometry (CH) and linear scan voltammetry (LSV). The influence of different interfering species and the effect of the operating temperature of 37 °C were also studied in order to simulate the operation of the sensor in the culture plate. The LSV technique made the sensor adaptable to undiluted media because the test time is short, compared with the CH technique, reducing the electrode fouling. The long-term stability of the sensors was also evaluated by testing different storage conditions. By storing the electrode at 4 °C, the sensor performance was not reduced for up to 21 days. The sensors were validated measuring H_2_O_2_ released by two different human bronchial epithelial cell lines (A549, 16HBE) and human primary bronchial epithelial cells (PBEC) grown in RPMI, MEM and BEGM/DMEM media. To confirm the results obtained with the sensor, the release of reactive oxygen species was also evaluated with a standard flow cytometry technique. The results obtained with the two techniques were very similar. Thus, the LSV technique permits using the proposed sensor for an effective oxidative stress quantification in different culture media and without dilution.

## 1. Introduction

The imbalance between the production of reactive oxygen species (ROS) from both endogenous and exogenous sources and antioxidant defense systems (including superoxide dismutase, catalase and glutathione peroxidase) leads to oxidative stress [1,2,3,4]. ROS production can be increased by various physiological or pathological conditions. If the antioxidant enzymes fail to rebalance this ROS production, an accumulation occurs, causing cell damage. This contributes to inflammation, aging, cancer and several chronic diseases, including chronic obstructive pulmonary disease (COPD) [5,6,7]. Cigarette smoke is a strong inducer of oxidative stress and represents the main risk factor for COPD. Accordingly, therapies aimed at reducing oxidative burden or increasing antioxidant defences are useful in COPD management exacerbations and in preserving lung functions [8].

ROS include superoxide anion, hydroxyl radical and H_2_O_2_, among others [9,10,11]. Their half-life time is very short due to their high reactivity, and they are difficult to quantify [12,13]. Among ROS, H_2_O_2_ has the longest half-life time as well as the ability to cross biological membranes and induce damage in the extracellular space [14,15,16]. Thus, the quantification of H_2_O_2_ in cellular supernatants appears to be a convenient way to monitor the oxidative status of the cell [17,18].

Nowadays, H_2_O_2_ quantification is carried out by different laboratory-based techniques such as fluorometric and colorimetric assays, nuclear magnetic resonance spectroscopy and liquid chromatography [19,20,21,22,23]. Among the drawbacks of these techniques are their high cost, long analysis time, requirements of highly skilled personnel, and more importantly, their inability to facilitate in situ analysis to provide real-time results. Indeed, a sample must be collected from the cell culture to quantify H_2_O_2_ released by cells. This makes it very challenging to continuously monitor oxidative stress in real-time during cell growth [24]. Electrochemical sensors are perfect candidates to minimize all these drawbacks because they can be applied for in situ and real-time analysis, while offering good performances in terms of sensitivity, selectivity and limit of detection [25,26,27,28,29,30,31,32,33,34,35]. To improve the performance of an electrochemical cell, the use of nanostructured electrodes ensures a high active surface area and promotes a high current density [36,37,38,39,40,41,42,43].

In our previous work, we developed and studied the features of a nanostructured electrode made of reduced graphene oxide (rGO) and gold nanoparticles (Au-NPs) for the chronoamperometric detection of H_2_O_2_ released by the human macrophages cell line, THP1, grown in a Roswell Park Memorial Institute (RPMI) 1640 medium [44]. This sensor could be used to measure H_2_O_2_ release from other types of cell cultures as well. Different media can be used to grow different cells or the same cells, and the same culture medium can be used to grow different cells [45,46,47]. Usually, a medium contains a very wide range of chemicals, stabilizers and nutrients [48,49,50,51] and can differ from the other in nutrients and growth factors [48,52,53,54]. The most commonly used media include RPMI 1640 [55], Eagle Minimum Essential Medium (MEM) [49], Dulbecco’s Modified Eagle Medium (DMEM) [56], Ham’s F-10 & F12 [57], Medium 199 [58], and Iscove’s Modified Dulbecco’s Medium (IMDM) [59]. Considering their complex composition, an influence on the electrochemical detection of H_2_O_2_ by the different growth media can be expected. For this reason, we have carried out a systematic investigation of the effect of the cell culture media on the electrochemical response of the sensor for the detection of H_2_O_2_ released by epithelial cells from central and distal airways. In recent years, a number of different nanostructures have been studied for the electrochemical detection of hydrogen peroxide, such as Pt NPs [60], Cu_6_(SC_7_H4NO)_6_ nanoclusters [61], MnO_2_ nanosheets [62] and Co_3_O_4_ nanowires [63]. Our sensor consists of an indium tin oxide/poly-ethylene terephthalate (ITO-PET) substrate modified by electrodeposited AuNPs and rGO. These active materials were selected because they were demonstrated to have improved detection sensitivity for H_2_O_2_ compared to sensors consisting of either AuNPs or rGO only [44,64,65,66,67].

To date, very few papers have reported on the influence of cell culture media on the performance of electrochemical sensors [68,69]. In this work, the effect of different culture media was studied in detail. Previously, AuNPs-rGO-based sensors were optimized and tested [44] in RPMI medium by chronoamperometry (CH). Using CH, it was necessary to dilute the sample with phosphate-buffered saline (PBS; pH 7.4) by 50% in volume to avoid fouling of the sensor surface, which could yield a low detection sensitivity. Starting from these results, the electrochemical sensor described in [44] was used here with the aim of performing a systematic investigation on the influence of the following parameters on the quantification of H_2_O_2_:

- Cell culture medium (MEM, DMEM, RPMI, Ham’s F12 and bronchial epithelial cell growth media (BEGM)/DMEM);
- Electrochemical quantification technique (CH and LSV);
- Medium dilution (undiluted vs. diluted in PBS (50% *v*/*v*));
- Operating temperature (25 °C and 37 °C);
- Interferents (uric acid, sodium chloride, lactic acid, glucose and HEPES);
- Storage condition (immersed in PBS or in deionized water and stored at 4 and 20 °C, sealed under vacuum and stored at 4 and 20 °C or stored at 4 and 20 °C in air).

Considering that the media have a very complex matrix, a fouling of the surface of the sensor is expected during its operation. To overcome this problem, a dilution of medium can be a solution. However, for a sensor that must operate in situ (in the culture plate during cell growth), a different approach is necessary, such as the use of a fast electrochemical technique that permits performing the analysis before the appearance of the fouling phenomena. For this reason, in this work, an LSV technique was also used, and the results were compared to those of conventional CH [70,71,72,73].

In addition, the sensor was validated quantifying the H_2_O_2_ released from different cell lines. In particular, human primary bronchial epithelial cells (PBEC), human bronchial epithelial cell line (16HBE) and adenocarcinoma alveolar basal epithelial cell line (A549) were cultured and stimulated with both pro-oxidant (cigarette smoke extract, (CSE) [74]) and antioxidant (resveratrol [75]) stimuli. The release of H_2_O_2_ release was also quantified with the electrochemical sensor, and results were compared with the data obtained by flow-cytometry using the same cells stained with Carboxy-H2DCFDA and MitoSOX Red probe, which detect intracellular ROS and mitochondrial superoxide, respectively. Both techniques revealed a significant increase in ROS in cells exposed to CSE. Resveratrol, an antioxidant molecule, reverted this effect. The AuNPs-rGO-based sensor offers high sensitivity and selectivity, a short response time (<60 s) and can be applied to real-time, in situ monitoring of H_2_O_2_ release.

## 2. Materials and Methods

### 2.1. Reagents

Flexible indium tin oxide/polyethylene terephthalate substrate (60 Ω cm^−2^) and graphene oxide (4 mgmL^−1^) were purchased from Sigma Aldrich (St. Louis, MO, USA) and Graphenea, respectively. The following reagents were purchased from AlphaAesar: KAuCl_4_, 2-propanol, sodium acetate, glacial acetic acid, PBS tablet, 30% (*v*/*v*) H_2_O_2_, 4-(2-hydroxyethyl)-1-piperazineethanesulfonic acid (HEPES), lactic acid, uric acid, sodium nitrate, sodium chloride, glucose.

MEM, RPMI-1640, DMEM, Fetal Bovine Serum (FBS), nonessential amino acids, L-glutamine, gentamicin, streptomycin and penicillin were obtained from Euroclone, while BEGM and Ham’s F12 Medium from Lonza Bioscience (see Supplementary Material for their composition). The probe 6-carboxy-2′,7′-dichlorodihydrofluorescein diacetate (H2DCFDA, C-2938) and MitoSOX™ Red mitochondrial superoxide indicator were bought from Life Technologies.

### 2.2. Sensor Fabrication and Electrochemical Detection of H_2_O_2_

The working electrode, made of AuNPs and rGO, was synthesized as previously described [44]. Briefly, the ITO-PET substrate was ultrasonically cleaned with pure iso-propanol and deionized water for 10 min. This electrode was then inserted into a home-made 3D printed cell (Figure S1), where the exposed active area of the electrode was 0.785 cm^2^. In this cell, a Pt wire was used as counter electrode while a saturated calomel electrode (SCE) as reference.

A Princeton Applied Research potentiostat/galvanostat (PARSTAT, mod. 2273) was used for both fabrication and characterization. AuNPs and rGO were potentiostatically co-deposited on the working electrode at −800 mV vs. SCE for 200 s in a solution containing 0.5 mgmL^−1^ GO and 0.5 mM KAuCl_4_ in acetate buffer (pH 5.4).

The effect of 5 different culture media (MEM, DMEM, Ham’s F12, RPMI and BEGM/DMEM (B/D)) on H_2_O_2_ quantification was evaluated. The AuNPs-rGO-based sensor was calibrated using both CH (at a constant potential of −800 mV vs. SCE) and LSV (in the potential range from +200 mV to −1200 mV, with a scan rate of 25 mV s^−1^). For CH tests, the medium was diluted with 50% of PBS (pH 7.4), while for LSV measurement, the sensor was calibrated in both diluted and undiluted media. Each calibration was carried out at least 3 times with 3 different electrodes. 

The effect of temperature on the sensor performance was also evaluated. Particularly, H_2_O_2_ was quantified at room temperature and 37 °C. This temperature was chosen because cells are conventionally cultured at 37 °C.

The sensor selectivity was studied using only LSV as an electrochemical technique because, in our previous work, it was already found that the electrode was selective also using CH. LSV was carried out in the presence of 0.5 mM of H_2_O_2_ and 5 mM of different interfering species (uric acid, sodium chloride, lactic acid, glucose and HEPES) that can be found in media.

The sensor stability was evaluated by measuring the output after 21 days of storing under different conditions (immersed in PBS or in deionized water and stored at 4 and 20 °C, sealed under vacuum and stored at 4 and 20 °C, stored at 4 and 20 °C in air).

### 2.3. Cell Culture Test

Cell line of immortalized human normal bronchial epithelial (16HBE) and cell line of lung adenocarcinoma (A549) (Interlab Cell Line Collection) were used.

16HBE were cultured in MEM with 10% fetal bovine serum (FBS) (heat-deactivated 56 °C, 30 min), 1% non-essential amino acids, 2 mM L-glutamine and 0.5% gentamicin [76]. A549 were cultured in DMEM with 10% FBS (heat deactivated at 56 °C, 30 min), streptomycin and penicillin, 1% nonessential amino acids and 2 mM L-glutamine. All cited components were obtained from Euroclone. 

16HBE and A549 were seeded in 6-well plates (500,000 cells/well) and the adherent cell culture monolayers were maintained in a humidified ambient, at 37 °C and with 5% CO_2_. Once reached the confluence (approximately 2 million cells/well), cells were stimulated with CSE at different concentrations (20% for 16HBE and 2.5% for A549) for 24 h. The 16HBE cells were also treated with resveratrol (40 μM), an antioxidant molecule, for 24 h in the presence or absence of CSE. 

Human primary bronchial epithelial cells (PBECs) (American Type Culture Collection (ATCC), Manassas V.A.; PCS-300-010) were also used. PBECs were differentiated using the air–liquid interface (ALI) culture. Cells were seeded on 0.4 μm pore sized 12-well transwell plates (40,000 cells/well) (Corning Costar, Cambridge, MA, USA) coated with human fibronectin (Santa Cruz Biotecnology, Dallas, TX, USA), bovine albumin fraction V (Sigma Aldrich, St. Louis, MO, USA) and PureCol^®^ (Advanced BioMatrix, Carlsbad, CA, USA) using B/D mixture (1:1, *v*/*v* BEGM/DMEM). The B/D medium contains 25 µM Hepes, bronchial epithelial cell growth supplement, 100 UmL^−1^ penicillin, 100 μgmL^−1^ streptomycin and 15 ngmL^−1^ retinoic acid receptor agonist EC23 (Tocris, Bristol, UK). Cells were cultured as submerged until confluence (approximately 500,000 cells/well), then the apical medium was removed and differentiated at ALI for 15 days. The differentiation of the cells into a more complex tissue containing different cell types is confirmed by the presence of cilia beating, by the mucus secretion and by the measurement of the trans-epithelial electrical resistance (TEER > 500 Ω cm^2^). After 15 days, PBECs were stimulated with CSE 20% for 24 h.

CSE was prepared by burning two cigarettes (3R4F-Kentucky—The Tobacco Research Institute, University of Kentucky) without any filter in 20 mL of PBS using a Watson–Marlow 323 E/D peristaltic pump (Rotterdam, The Netherlands). The solution was filter-sterilized (0.22 μm pore filter) and considered to be 100% of CSE. This was further diluted in the cell culture medium to reach the specific concentration for each experiment.

Flow cytometry was used to measure intracellular ROS and mitochondrial superoxide. After the stimulation, the cells were harvested and stained with 1 μM of 6-carboxy-2′, 7′-dichlorodihydrofluorescein diacetate (Carboxy-H2DCFDA) probe to measure intracellular ROS (30 min, ambient temperature) and with 3 μM of MitoSOX™ Red probe, which is specific for mitochondrial superoxide (15 min, at 37 °C) [77,78]. The flow cytometer CytoFLEX (Beckman Coulter, Brea, CA, USA) was used in these assays. The results were expressed as the mean of fluorescence intensity (MFI).

## 3. Results and Discussion

The sensor tested in this work consists of AuNPs and rGO that were co-electrodeposited on ITO/PET substrate. In this sensor, AuNPs and rGO act as active materials for the quantification of H_2_O_2_, while ITO acts only as conducting material. Due to its poor electrocatalytic properties, ITO does not contribute to the sensitivity of the sensor but simply makes the PET substrate conductive, thus making possible the electrodeposition of Au and rGO and the subsequent electrochemical characterization tests. Thus, the sensing material consists of only Au-NPs (about 33 nm) and r-GO flakes (about 10 × 18 μm). According to the results obtained in [44], AuNPs-rGO-based sensors tested in RPMI as medium diluted with 50% of PBS displayed for the detection of H_2_O_2_ a limit of detection (LOD) of 6.55 µM and an average sensitivity of 0.064 μAμM^−1^cm^−2^. In the previous study [44], we also demonstrated that using CH, the dilution of medium (50%) is necessary to have good sensitivity because, in medium alone, a decrease in sensitivity of about an 80% was observed due to fouling phenomena. In this work, the same experiments were carried out using other culture media. In particular, in each test, at the start of CH PBS alone was present in the cell. After the stabilization of the current, an equal volume of medium was added to have a dilution of 50%. The addition of the medium causes a spike in the current due to the instantaneous change in the solution composition at the electrode interface. After the transient, the current stabilizes, and the value is used as the blank current. In subsequent tests, the culture medium injected into the cell contains different amounts of H_2_O_2_. This causes a variation in the current, which depends on the concentration of H_2_O_2_.

**Figure 1:**
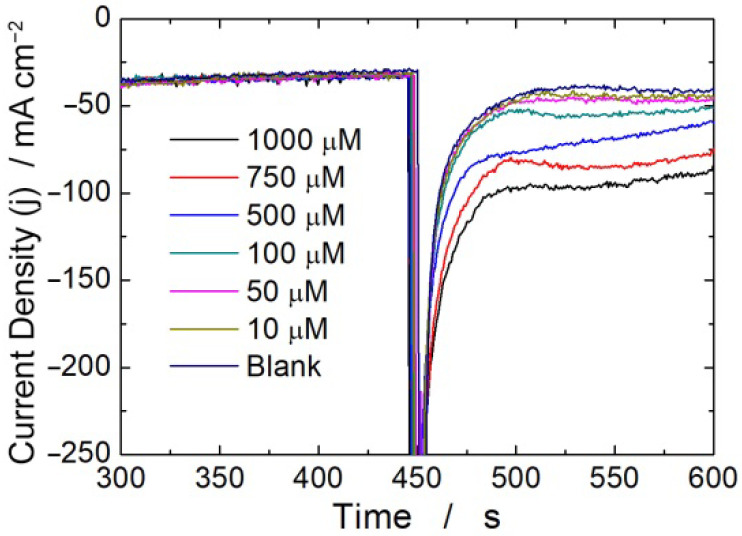
Effect of increasing H_2_O_2_ concentration on CH experiment carried out at −800 mV vs. SCE using diluted MEM medium.

**Figure 2:**
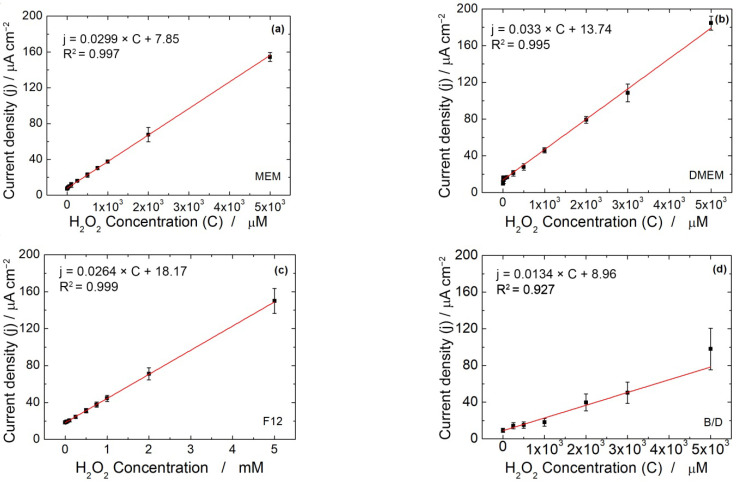
Calibration line obtained with CH at −800 mV vs. SCE in (**a**) diluted MEM, (**b**) diluted DMEM, (**c**) diluted Ham’s F12 and (**d**) diluted B/D medium (*n* = 3).

Figure 1 (see also Figure S2) shows the effect of increasing H_2_O_2_ concentration on the CH experiments carried out using diluted MEM (1:1 *v*/*v* in PBS) as the solution. As expected, the higher the H_2_O_2_ concentration, the higher the output current density. A similar behavior was obtained in the other tested media. Considering that sensor calibration depends on the current value, careful and objective evaluations must be made. Thus, to build the calibration line, the output current density was selected based on the slope of the i-t curve. In particular, the current density corresponding to a slope equal to or lower than 50 nAs^−1^ was selected. Operating in this way, the value of the current used for the calibration of the sensor is independent of the operator’s choice, and above all, it is independent of the precise time in which the measurement is made. With this procedure, all the i-t curves were processed, including that obtained for the blank. The current measured for the blank was subtracted from the values obtained by varying the concentration of H_2_O_2_, and the obtained data were used to construct the calibration lines, as shown in Figure 2.

Figure 2 shows the calibration plots based on the CH signal obtained in different culture media at −800 mV vs. SCE. As can be observed, the electrode sensitivity (estimated by the slope of each linear calibration expression) was affected by the medium composition. Chronoamperometric detection of H_2_O_2_ maintains a similar sensitivity with MEM, DMEM and Ham’s F12, while the sensitivity dropped down to 0.0138 µAµM^−1^cm^−2^ using B/D medium. For each medium, the LOD was calculated by measuring the standard deviation of the blank using the following equation [79]:

LOD = 3.3 × SD/S(1)

where SD is the standard deviation of the blank, and S is the electrode sensitivity. The standard deviation of the blank using MEM, DMEM and RPMI was very similar (ranging from 0.128 to 0.157 µAcm^−2^), while it was much higher using Ham’s F12 and B/D (0.406 and 0.51 µAcm^−2^).

In Table 1, the analytical parameters estimated using CH carried out in diluted culture media are tabulated.

From the results reported in Table 1, it can be concluded that the best medium for chronoamperometry quantification of H_2_O_2_ is the RPMI. This result is attributable to the different media compositions.

**Figure 3:**
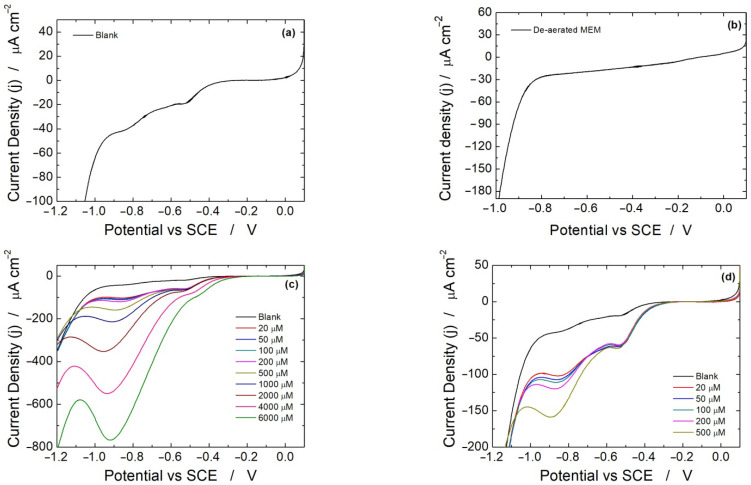
Linear scan voltammetry experiments carried out in: (**a**) MEM blank solution, (**b**) de-aerated MEM blank solution, (**c**,**d**) aerated MEM blank solution with different H_2_O_2_ concentrations. In all experiments, undiluted MEM was used (*n* = 3).

The same experiments were carried out using LSV as the electrochemical technique. The potential was scanned from +200 to −1200 mV vs. SCE at a scan rate of 25 mV/s. The linear scan voltammograms obtained in a blank solution and in MEM de-aerated using a continuous N_2_ flux are shown in Figure 3a,b, respectively. Figure 3b shows a featureless voltammogram, whereas Figure 3a depicts two peaks at −0.5 and −0.8 V, which are likely to be related to dissolved oxygen.

The first peak at −0.5 V is attributed to the reduction in dissolved oxygen to H_2_O_2_, following Equation (2) [80,81]

(2) $$O_{2}+2H_{2}O+2e^{-}\to H_{2}O_{2}+2{\mathrm{OH}}^{-}$$

The second peak at −0.8 V is related to the reduction in H_2_O_2_ to water, following Equation (3) [82,83]:

(3) $$H_{2}O_{2}+2H^{+}+2e^{-}\to 2H_{2}O$$

**Figure 4:**
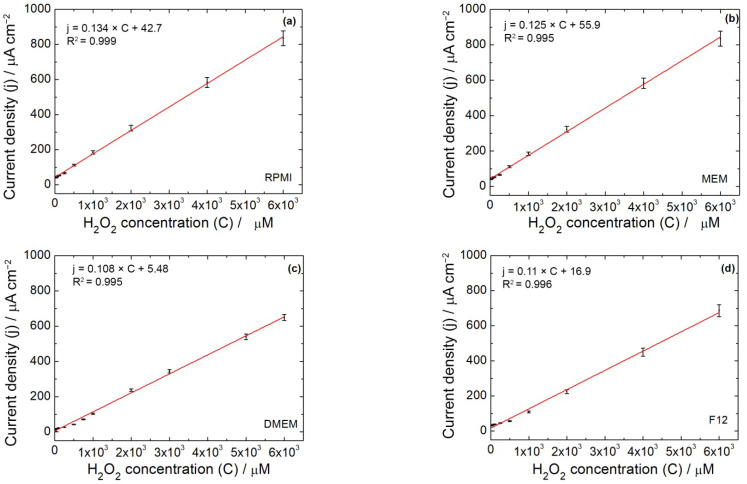

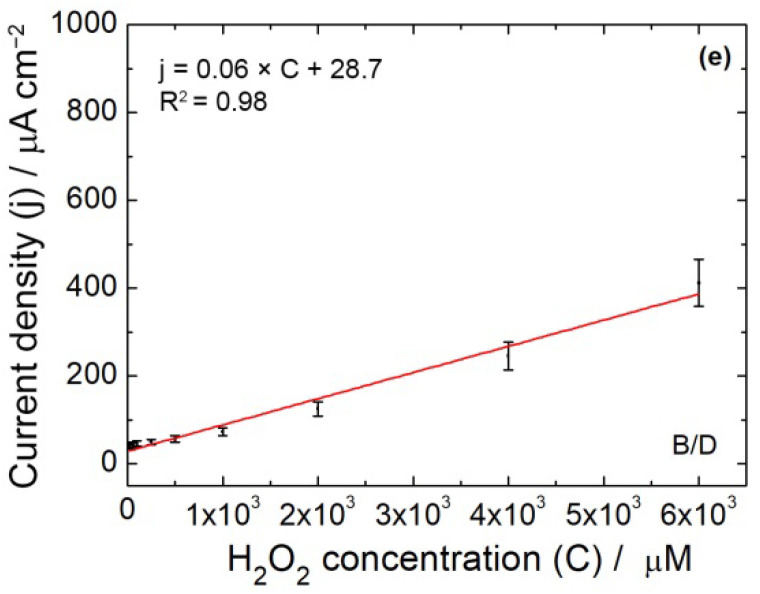
Calibration line obtained with LSV in: (**a**) RPMI alone, (**b**) MEM alone, (**c**) DMEM alone, (**d**) Ham’s F12 alone, (**e**) B/D alone (*n* = 3).

Thus, even in the absence of H_2_O_2_, the presence of dissolved oxygen at about −400 mV generates a small amount of H_2_O_2_ that is then revealed at more cathodic potentials [84]. When different concentrations of H_2_O_2_ are spiked into the solution, the corresponding voltammograms are displayed in Figure 3c. In these voltammograms, the first peak at −0.4 V does not increase in intensity, while the peak at −0.8 V increases with H_2_O_2_ concentration [85]. To illustrate this point more clearly, only linear scan voltammograms for low H_2_O_2_ concentration are shown in Figure 3d. These results are a further confirmation that the peak at about −400 mV is attributable to the electro-generation of H_2_O_2_ from dissolved oxygen reduction. According to Nernst’s equation, at high H_2_O_2_ concentration, the peak initially located at −0.8 V shifts to −0.9 V. A similar behavior was observed using both diluted and undiluted culture media. Figure 4 shows the obtained calibration lines using the media alone. The sensor performances are reported in Table 2. Interestingly, the sensitivity is much higher using LSV compared to CH (Table 1). For all studied media, the sensitivity increases by a factor of 3–4.

Using MEM, RPMI and DMEM, the effect of dilution is almost negligible (Table 2), while for Ham’s F12 and B/D, there is still an increase in sensitivity after dilution. Furthermore, in those media, better results were obtained with LSV compared to CH. This effect could be attributed to the fastness of the LSV compared to CH. In fact, the whole LSV experiments lasted less than 60 s, while CH experiments lasted for at least 600 s, due to the requirement for signal stabilization. During stabilization, H_2_O_2_ reacts at the electrode surface along with all the other chemical species present in the culture media. This may strongly affect the signal output due to a bio-fouling effect, especially with more complex mediums such as Ham’s F12 or B/D. Thus, the AuNPs-rGO-based sensor can work with every kind of medium with both LSV or CH measurements but, depending on the medium and the expected H_2_O_2_ concentration, it is necessary to use LSV or CH as the electrochemical technique to have a high sensitivity. In particular, the sensor can detect low concentrations of H_2_O_2_ in RPMI, MEM and DMEM medium with both LSV and CH, while for B/D, CH can be used when high concentrations of H_2_O_2_ are expected. LSV must be used to detect low concentrations of H_2_O_2_ (Table 1 and Table 2). From these results, it can be concluded that the proposed sensor is adaptable for use in different media.

**Figure 5:**
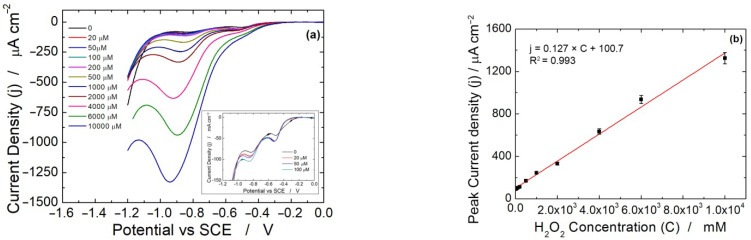
(**a**) LSV experiments at increasing H_2_O_2_ concentration and (**b**) corresponding calibration line. The tests were performed in pure MEM and at 37 °C (*n* = 3).

Considering that the final goal of our research is to use the sensor directly on the plate during the culture of cells, the influence of operating temperature was also studied. Particularly, as the cell lines are cultured in incubators at a controlled temperature of 37 °C, the electrode was also tested at this temperature in order to simulate the operation in the plate [86]. Figure 5a,b shows the linear scan voltammograms obtained at 37 °C and the corresponding calibration line, respectively. The output current density increases with temperature, while electrode sensitivity is almost constant. In fact, a value of 0.127 μAμM^−1^cm^−2^ was obtained compared to 0.125 μAμM^−1^cm^−2^ obtained at room temperature (Table 2). The higher cathodic current density in Figure 5b, compared to that of Figure 4b, is expected at higher temperature due to the more favorable kinetics [87,88]. Thus, the sensor can also operate in the plate during the cell growth.

**Figure 6:**
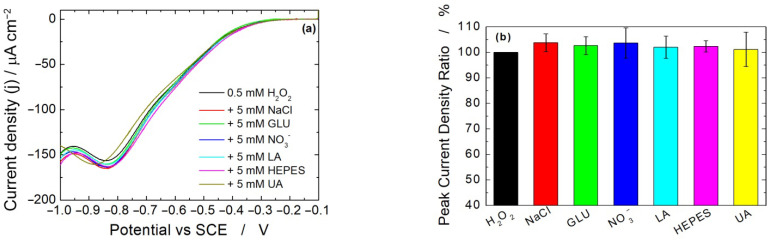
(**a**) LSV experiments carried out in MEM alone in the presence of 0.5 mM H_2_O_2_ and 5 mM of interfering species and (**b**) corresponding interference of each chemical on sensor output (*n* = 3).

To fully characterize the electrode features for H_2_O_2_ quantification, a selectivity test was carried out towards different chemicals that could be found in the different culture media or that could be generated by the different cell lines. Particularly, 5 mM of the interfering species (sodium chloride, sodium nitrate, glucose, lactic acid, HEPES and uric acid) were added to the solution containing 0.5 mM of H_2_O_2_. This is a conservative condition due to the high concentration of interfering species compared to the concentration of H_2_O_2_ ([H_2_O_2_]/[interfering] = 10) [89]. Figure 6 shows the results both as LSV curves (Figure 6a) and as a ratio between the peak current measured in the absence and in the presence of the interfering species (Figure 6b). In all conditions, the interference is negligible (lower than 5%) in terms of both current intensity and potential. This high selectivity of the sensor is expected because the potential at −0.8 V is way off the redox peaks of interferences. Only in the case of uric acid was a very small shift in potential value (−30 mV) observed. This interference was probably due to the different pH of the solution. In fact, uric acid must be solubilized in a 0.1 M KOH due to its low solubility, and thus, its injection in the electrochemical cell can modify the solution pH [90]. Similar behavior was found in the other media.

Finally, to study the stability of the AuNPs-rGO-based sensors over time, the effect of different storage methods was evaluated by measuring the current density for 5 mM H_2_O_2_ before and after 21 days of storage. In particular, electrodes were stored in the following conditions:

- Immersed in PBS or in deionized water and stored at 4 and 20 °C;
- Sealed under vacuum and stored at 4 and 20 °C;
- Stored at 4 and 20 °C in air.

The results are summarized in Table 3. All the storage conditions with the electrode immersed in liquid solutions (PBS or deionized water) were found to be almost destructive for the electrode, with a signal reduction ≥20%. A similar result was found storing the electrode in air at room temperature, with a signal reduction of 36%.

The best storing conditions were found to be at 4 °C in air and vacuum at both 4 and 20 °C. In these conditions, the change in current density was lower than 10%. Considering that the sensor has a reproducibility of about 5% (see Figure 4), these storage methods are suitable for storing it without appreciable deterioration in performance. Thus, it can be concluded that the sensors stored in these conditions are stable for at least 21 days after their production. This result agrees with other studies [91,92], where the same storage method was used for similar sensors (based on AuNPs and rGO but on a different substrate).

**Figure 7:**
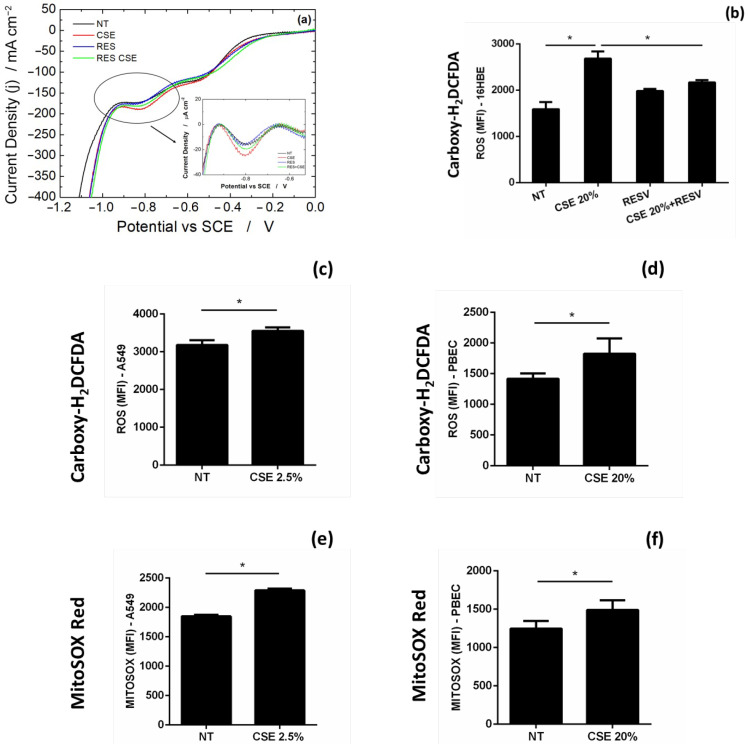
(**a**) LSV experiments with 16HBE cell line and ROS detection by flow cytometry using Carboxy-H2DCFDA probe in (**b**) 16HBE, (**c**) A549, and (**d**) PBEC and using Mitosox Red probe in (**e**) A549, and (**f**) PBEC. In the inset of (**a**) the LSV curves after baseline subtraction. * *p* value < 0.05 Unpaired *t*-test (*n* = 3).

The AuNPs-rGO-based sensor was used to quantify the H_2_O_2_ released by A549, 16HBE and PBEC cells grown in RPMI, MEM and B/D, respectively. Considering the higher sensitivity obtained with LSV as the electrochemical technique, these samples were analyzed using this technique. A549 and PBEC were tested in two different conditions: untreated cells (NT) and treated with CSE as a pro-oxidant stimulus. 16HBE cell line was tested after the treatment with CSE and with an antioxidant molecule resveratrol (RES) and a combination of pro- and antioxidant stimula (RES + CSE). Figure 7a shows the linear scan voltammograms obtained in 16HBE cells. Particularly, Figure 7a shows the whole LSV curves obtained with 16HBE cell line, while background-subtracted voltammograms are shown in the inset. As expected, in all the studied cell lines, CSE treatment led to an increase in H_2_O_2_ production, while the treatment with RES, tested only in 16HBE cells, thanks to its antioxidant action, reverted the CSE-induced increase in H_2_O_2_ release. The results obtained in all experiments are summarized in Table 4.

To confirm the results obtained with the sensor, we used the same cells in which we tested the release of H_2_O_2_ in the culture medium to evaluate cellular oxidative stress assessing the production of intracellular ROS and mitochondrial superoxide after stimulation with cigarette smoke (pro-oxidant) and RES (antioxidant), the latter only in 16HBE. For this purpose, we used Carboxy-H2DCFDA and Mitosox Red probe, commonly used in research laboratories, which bound the intracellular ROS and mitochondrial superoxide, respectively, and evaluated their expression by flow cytometry. As Figure 7 shows, treating 16HBE (b), A549 (c, e) and PBEC (d, f) with CSE increases the production of intracellular ROS and mitochondrial superoxide, while RES reduces cigarette smoke-induced oxidative stress in 16HBE (b). These results are in line with those obtained with AuNPs-rGO-based sensors, confirming the possibility of using the latter as a replacement for more expensive and time-consuming laboratory techniques for monitoring cellular oxidative stress.

## 4. Conclusions

In this work, we have shown the effect of different parameters on the quantification of H_2_O_2_ using a AuNPs-rGO-based sensor. In particular, the detection was performed using different cell culture media (diluted and undiluted), different electrochemical techniques (CH and LSV) and temperature operations (25 and 37 °C). Furthermore, the effect of different interferents and storage conditions was studied. Using CH, the AuNPs-rGO-based sensor showed a sensitivity ranging from 0.033 to 0.064 μAμM^−1^cm^−2^ using MEM, DMEM or RPMI as medium, while the sensitivity decreased using Ham’s F12 or B/D. The LOD was about 10 µM with RPMI, MEM and DMEM, while it increased to up to 50–80 µM in Ham’s F12 and B/D. LSV was also used to quantify H_2_O_2_, and results showed a higher sensitivity (about four times) in all the studied medium, with a consistent decrease in LOD. The selectivity test showed an excellent anti-interference property of the AuNPs-rGO-based sensor. The effect of electrode storage for 21 days was also studied in different conditions. The results showed that the best storage method consists of in storage at 4 °C in air and in the dark. In these conditions, the current density changed by only 2% after 21 days, confirming the long-term stability of AuNPs-rGO-based sensor. To use the electrode directly in the cell culture plate, the effect of operating temperature was studied, and a negligible effect was found, showing an increase in current density with a constant sensitivity. 

The sensor was used to quantify H_2_O_2_ in different cell cultures. In particular, the H_2_O_2_ released from A549, 16HBE and PBEC cells exposed to pro-oxidant and antioxidant treatments was measured. The sensor was able to quantify the variation of H_2_O_2_ released from different cell types after different treatments. These results were validated by flow cytometry, a technique providing a quantitative measure of intracellular oxidative stress. Thus, the AuNPs-rGO-based sensor can be effectively used to quantify oxidative stress in cell culture medium in a fast, easy, cheap, reproducible, and sensitive way.

## Figures and Tables

**Table 1:** Features of the AuNPs-rGO-based sensors using CH at −800 mV vs. SCE as the electrochemical technique in different culture media diluted with PBS.

Medium	Sensitivity µAµM^−1^cm^−2^	Limit of Detection µM	Linear Range µM
Diluted RPMI	0.064	6.55	25–5000
Diluted MEM	0.0299	13.05	10–5000
Diluted DMEM	0.033	15.37	10–5000
Diluted Ham’s F12	0.0264	51.61	100–5000
Diluted B/D	0.0138	80.14	250–5000

**Table 2:** Performance of the AuNPs-rGO-based sensors using LSV as the electrochemical technique in different culture media diluted and not diluted with PBS.

Medium	Sensitivity µAµM^−1^cm^−2^	Limit of Detection µM	Linear Range µM
Pure RPMI	0.134	3.12	20–6000
Diluted RPMI	0.134	3.12	20–6000
Pure MEM	0.125	4.57	20–6000
Diluted MEM	0.125	4.57	20–6000
Pure DMEM	0.108	5.7	20–6000
Diluted DMEM	0.105	5.8	20–6000
Pure Ham’s F12	0.11	16.77	50–6000
Diluted Ham’s F12	0.08	23.06	50–6000
Pure B/D	0.06	26.71	100–6000
Diluted B/D	0.11	15.2	50–6000

**Table 3:** Stability of the AuNPs-rGO-based sensors after 21 days of storage.

Sample	Current Density µAcm^−2^	Difference %
Fresh electrode	240 ± 12	0%
Immersed in deionized water at 4 °C	189 ± 8.5	−21.2%
Immersed in deionized water at 20 °C	212 ± 11.7	−12%
Immersed in PBS at 4 °C	157 ± 7.5	−35%
Immersed in PBS at 20 °C	171 ± 8.9	−29%
Stored in air at 4 °C	245 ± 9.8	+2%
Stored in air at 20 °C	153 ± 9.2	−36%
Vacuum at 4 °C	259 ± 14.2	+7.9%
Vacuum at 20 °C	222 ± 9.3	−7.5%

**Table 4:** Real samples analysis tested using AuNPs-rGO-based sensors with LSV.

Sample	NT (µM)	CSE (µM)	RES (µM)	RES + CSE (µM)
16 HBE-MEM	10.63 ± 1.28	41.5 ± 11.8	9.7 ± 2.1	15.58 ± 3.58
A549-DMEM	12.92 ± 1.04	15.8 ± 1.15	-	-
PBEC-B/D	42.6 ± 7.1	129 ± 21.5	-	-

## Data Availability

Not applicable.

## Supplementary Materials

The following supporting information can be downloaded at: https://www.mdpi.com/article/10.3390/mi13101762/s1, Tables with media composition. Figure S1: Photo of the 3D printed cell. Figure S2: Effect of increasing H_2_O_2_ concentration on CH experiment carried out at −800 mV vs. SCE using diluted MEM medium.

## Abbreviations

AuNPs-rGO	sensor
16HBE	human normal bronchial epithelial cell line
A549	lung adenocarcinoma cell line
ALI	air-liquid interface culture
AuNPs	gold nanoparticles
BEGM	bronchial epithelial cell growth media (BEGM)
B/D	BEGM/DMEM (1:1) medium
Carboxy-H2DCFDA	6-carboxy-2′,7′-dichlorodihydrofluorescein diacetate
CH	chronoamperometry
COPD	chronic obstructive pulmonary disease
CSE	cigarette smoke extract
DMEM	Dulbecco’s modified eagle medium (DMEM)
FBS	fetal bovine serum (FBS)
HEPES	4-(2-hydroxyethyl)-1-piperazineethanesulfonic acid
ITO-PET	indium tin oxide/polyethylene terephthalate substrate
LOD	limit of detection
LSV	linear scan voltammetry
MEM	Eagle’s minimum essential medium (MEM),
MFI	mean of fluorescence intensity
PBECs	human primary bronchial epithelial cells
PBS	Phosphate-buffered saline
RES	resveratrol
rGO	reduces graphene oxide
ROS	reactive oxygen species
SCE	saturated calomel electrode
RPMI-1640	Roswell Park Memorial Institute-1640 medium,
TEER	trans-epithelial electrical resistance
