# Supplementary material for: Electrochemical Quantification of H2O2 Released by Airway Cells Growing in Different Culture Media
Source: Micromachines (Basel). 2022 Oct 18;13(10):1762. doi: 10.3390/mi13101762 (PMC9611932; doi:10.3390/mi13101762)
Supplement: Supplementary file 1 [file micromachines-13-01762-s001.zip › micromachines-1964688-supplementary.pdf]

Electrochemical Quantification of H_2O_2 Released by Airways Cells Growing in Different Culture Media

Bernardo Patella [1,*], Serena Di Vincenzo [2,*], Claudio Zanca[1], Luciano Bollaci [1], Maria Ferraro [2], Maria Rita Giuffrè [3], Chiara Cipollina [2,3], Maria Giuseppina Bruno [1], Giuseppe Aiello [1], Michele Russo [4], Rosalinda Inguanta [1] and Elisabetta Pace [2]

[1] Department of Engineering, University of Palermo, 90128 Palermo, Italy
[2] Institute of Traslational Pharmacology (IFT), National Research Council of Italy (CNR), 90146 Palermo, Italy
[3] Ri.MED Foundation, 90146 Palermo, Italy
[4] Dipietro Group, 96010 Melilli, Italy
* Correspondence: bernardo.patella@unipa.it (B.P.); serena.divincenzo@ift.cnr.it (S.D.V.)

BEBM™ Basal Medium 500 ml

- Orange Cap Vial with BPE, 2.00 mL
- Lilac Cap Vial with Insulin, 0.50 mL
- Natural Cap Vial with Hydrocortisone, 0.50 mL
- Red Cap Vial with GA-1000, 0.50 mL
- Amber Vial with Retinoic Acid, 0.50 mL
- Natural Cap Vial with Transferrin, 0.50 mL
- Amber Vial with Triiodothyronine, 0.50 mL
- Amber Vial with Epinephrine, 0.50 mL
- Green Cap Vial with hEGF, 0.50 mL

EuroClone®

serving science through innovation

Composition of DMEM High Glucose w Sodium Pyruvate w/o L-Glutamine

Code: ECB7501L

Amino acids :	
Glycine	30 mg/l
L-Arginine+HCl	84 mg/l
L-Cystine+2HCl	62.6 mg/l
L-Histidine+HCl+H_20	42 mg/l
L-Isoleucine	104.8 mg/l
L-Leucine	104.8 mg/l
L-Lysine+HCl	146.20 mg/l
L-Methionine	30 mg/l
L-Phenylalanine	66 mg/l
L-Serine	42 mg/l
L-Threonine	95.20 mg/l
L-Tryptophan	16 mg/l
L-Tyrosine	72 mg/l
L-Valine	93.6 mg/l
**Inorganic salt :**	
Calcium chloride	200 mg/l
Ferric nitrate Nonahydrate	0.1 mg/l
Magnesium sulfate+$7H_2O$	200 mg/l
Potassium chloride	400 mg/l
Sodium Phosphate Monobasic+H_2O	125 mg/l
Sodium bicarbonate	3700 mg/l
Sodium chloride	6400 mg/l

Vitamins :	
Choline chloride	4 mg/l
D-Ca pantothenate	4 mg/l
Folic acid	4 mg/l
I-inositol	7 mg/l
Nicotinamide	4 mg/l
Pyridoxal + HCl	4 mg/l
Riboflavin	0.4 mg/l
Thiamine+HCl	4 mg/l
**Other components :**	
D-Glucose	4500 mg/l
Phenol red	15.34 mg/l
Pyruvic Acid.Na	110 mg/l

ed1/0214/691_ ECB7501L

EuroClone S.p.A.
Via Figino 20/22, 20016 Pero (MI) - Italy
Phone: +39.02.38195.1 - Fax +39.02.38101465
e-mail: info@euroclone.it

CELBIO BIOAIR CELL BIOLOGY

www.euroclone.it

EuroClone S.p.A. has a Quality System certified in compliance with UNI EN ISO 9001:2008 and NF EN ISO 13485:2004

EuroClone®

serving science through innovation

Composition of Ham's F12 w/o L-Glutamine

Code: ECB7502L

Amino acids :	
Glycine	7.51 mg/l
L-Alanine	9 mg/l
L-Arginine+HCl	211 mg/l
L-Asparagine+H_20	15.01 mg/l
L-Aspartic acid	13.3 mg/l
L-Cysteine+HCl+H_20	35 mg/l
L-Glutamic acid	14.7 mg/l
L-Histidine+HCl+H_20	20.96 mg/l
L-Isoleucine	3.94 mg/l
L-Leucine	13.1 mg/l
L-Lysine+HCl	36.5 mg/l
L-Methionine	4.48 mg/l
L-Phenylalanine	4.96 mg/l
L-Proline	34.5 mg/l
L-Serine	10.5 mg/l
L-Threonine	11.9 mg/l
L-Tryptophan	2.04 mg/l
L-Tyrosine + 2Na + $2H_20$	7.78 mg/l
L-Valine	11.7 mg/l
**Inorganic salt :**	
Calcium chloride Dihydrate	44.1 mg/l
Cupric sulfate pentahydrate	0.0025 mg/l
Ferrous sulfate Heptahydrate	0.834 mg/l
Magnesium chloride Hexahydrate	123 mg/l
Potassium chloride	224 mg/l
Sodium bicarbonate	1176 mg/l
Sodium chloride	7599 mg/l
Sodium phosphate dibasic anhydrous	142.04 mg/l
Zinc sulfate Heptahydrate	0.863 mg/l
**Vitamins :**	
Choline chloride	13.96 mg/l
D-Biotin	0.0073 mg/l
D-Ca pantothenate	0.48 mg/l
Folic acid	1.32 mg/l
Myo-inositol	18 mg/l
Nicotinamide	0.037 mg/l
Pyridoxine+HCl	0.062 mg/l
Riboflavin	0.038 mg/l
Thiamine+HCl	0.34 mg/l
Vitamine B12	1.36 mg/l
**Other components :**	
D-Glucose	1802 mg/l
Hypoxanthine	4.08 mg/l
Linoleic acid	0.084 mg/l
Phenol red	1.3 mg/l
Putrescine+2HCL	0.161 mg/l
Sodium pyruvate	110 mg/l
Thioctic acid	0.21 mg/l
Thymidine	0.73 mg/l

ed1/0214/691_ ECB7502L

EuroClone S.p.A.
Via Figino 20/22, 20016 Pero (MI) - Italy
Phone: +39.02.38195.1 - Fax +39.02.38101465
e-mail: info@euroclone.it

CELBIO BIOAIR® CELL BIOLOGY

www.euroclone.it

EuroClone®
serving science through innovation

Composition of MEM w/ Earle's Salts w/o L-Glutamine

Code: ECB2071L

Amino acids :	
L-Arginine+HCl	126 mg/l
L-Cystine+2HCl	31.3 mg/l
L-Histidine+HCl+H_2O	42 mg/l
L-Isoleucine	52 mg/l
L-Leucine	52 mg/l
L-Lysine+HCl	72.5 mg/l
L-Methionine	15 mg/l
L-Phenylalanine	32 mg/l
L-Threonine	48 mg/l
L-Tryptophan	10 mg/l
L-Tyrosine $2Na_2$+H_2O	51.9 mg/l
L-Valine	46 mg/l
**Inorganic salt :**	
Calcium chloride Dihydrate	265 mg/l
Magnesium sulfate Anhydrous	97.67 mg/l
Potassium chloride	400 mg/l
Sodium Phosphate Monobasic Anydrous	122 mg/l
Sodium bicarbonate	2200 mg/l
Sodium chloride	6800 mg/l

Vitamins :	
Choline chloride	1 mg/l
D-Ca pantothenate	1 mg/l
Folic acid	1 mg/l
Myo-inositol	2 mg/l
Nicotinamide	1 mg/l
Pyridoxine + HCl	1 mg/l
Riboflavin	0.1 mg/l
Thiamine+HCl	1 mg/l
**Other components :**	
D-Glucose	1000 mg/l
Phenol red	11 mg/l

ECB2071_Ed2_0218_691

CELL BIOLOGY

www.euroclone.it

Euroclone S.p.A.
Via Figino 20/22, 20016 Pero (MI) - Italy Phone:
+39.02.38195.1 - Fax +39.02.33913713
e-mail: info@euroclone.it

EuroClone®

serving science through innovation

RPMI 1640

Code: ECB9006

Amino acids :	
Glycine	10 mg/l
L-Arginine	200 mg/l
L-Asparagine + H_2O	50 mg/l
L-Aspartic acid	20 mg/l
L-Cystine Dihydrochloride	65,2 mg/l
L-Glutamic acid	20 mg/l
L-Histidine	15 mg/l
L-Hydroxy-L-proline	20 mg/l
L-Isoleucine	50 mg/l
L-Leucine	50 mg/l
L-Lysine+HCl	40 mg/l
L-Methionine	15 mg/l
L-Phenylalanine	15 mg/l
L-Proline	20 mg/l
L-Serine	30 mg/l
L-Threonine	20 mg/l
L-Tryptophan	5 mg/l
L-Tyrosine disodium salt dihydrate	28,8 mg/l
L-Valine	20 mg/l

Inorganic salt :	
Calcium Nitrate Tetrahydrate	100 mg/l
Magnesium sulfate anhydrous	48,8 mg/l
Potassium chloride	400 mg/l
Sodium bicarbonate	2000 mg/l
Sodium chloride	6000 mg/l
Sodium phosphate dibasic anhydrous	2000 mg/l
**Vitamins :**	
Choline chloride	3 mg/l
p-Amino Benzoic Acid	1 mg/l
D-Biotin	0.2 mg/l
D-Ca pantothenate	0.25 mg/l
Folic acid	1 mg/l
Myo-inositol	35 mg/l
Nicotinamide	1 mg/l
D-Pantothenic acid	0,25 mg/l
Pyridoxine+HCl	1 mg/l
Riboflavin	0.2 mg/l
Thiamine+HCl	1 mg/l
Vitamine B12	0.005 mg/l
**Other components :**	
D-Glucose	2000 mg/l
L-Glutathione reduced	1 mg/l
Phenol red	5,3 mg/l

ECB9006_Ed1_0218_691

CELL BIOLOGY

Euroclone S.p.A.
Via Figino 20/22, 20016 Pero (MI) - Italy Phone: +39.02.38195.1 - Fax +39.02.33913713
e-mail: info@euroclone.it

www.euroclone.it

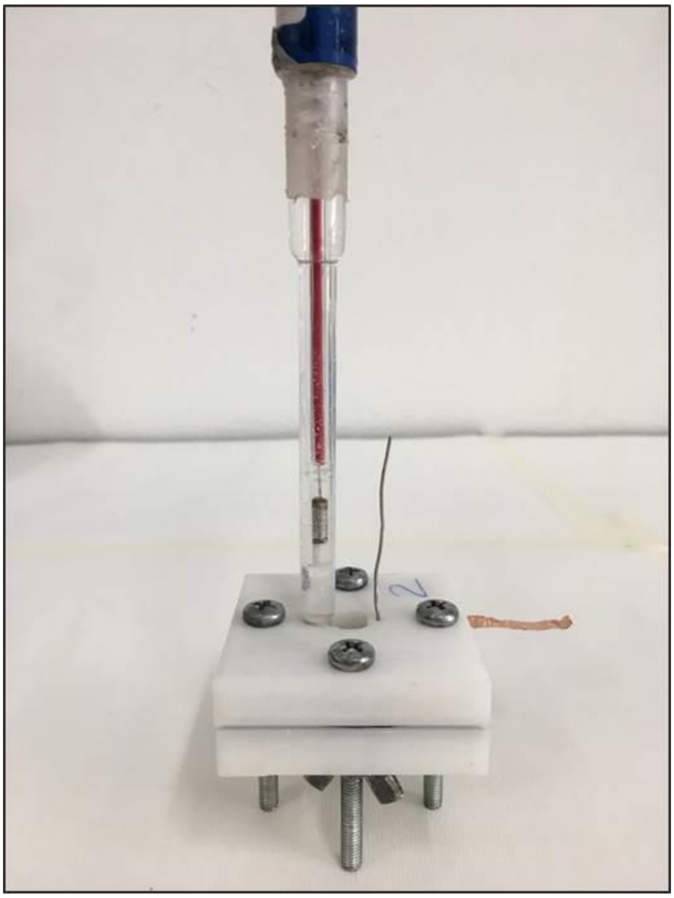

Figure S1. Photo of the 3D printed cell

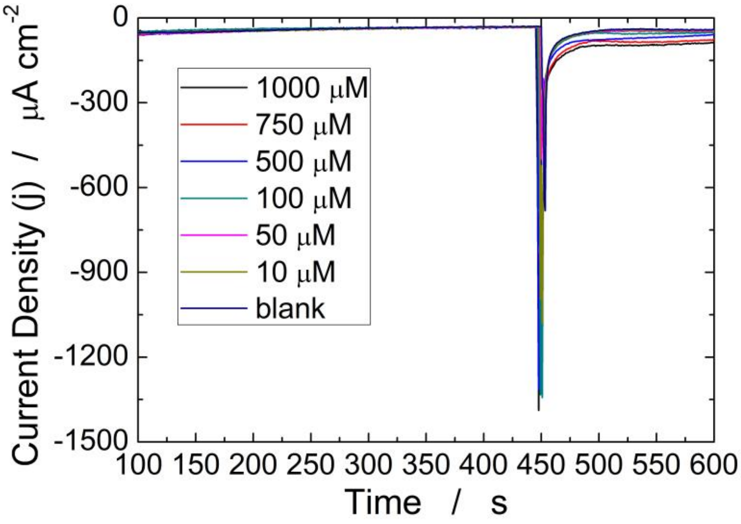

Figure S2. Effect of increasing H_2O_2 concentration on CH experiment carried out at -800 mV vs SCE using diluted MEM medium
